# Antibacterial and Antifungal Activity of Three Monosaccharide Monomyristate Derivatives

**DOI:** 10.3390/molecules24203692

**Published:** 2019-10-14

**Authors:** Jumina Jumina, Mutmainah Mutmainah, Bambang Purwono, Yehezkiel Steven Kurniawan, Yana Maolana Syah

**Affiliations:** 1 Department of Chemistry, Faculty of Mathematics and Natural Sciences, Universitas Gadjah Mada, Yogyakarta 55281, Indonesia; mutmainah@mail.ugm.ac.id (M.M.); purwono.bambang@ugm.ac.id (B.P.); yehezkiel.steven.k@mail.ugm.ac.id (Y.S.K.); 2 Faculty of Mathematics and Natural Sciences, Institut Teknologi Bandung, Bandung 40132, Indonesia; yana@chem.itb.ac.id

**Keywords:** monosaccharide, myristate, esterification, antibacterial, antifungal

## Abstract

Microbial infections remains a serious challenge in food industries due to their resistance to some of the well-known antibacterial and antifungal agents. In this work, a novel monomyristoyl ester (fructosyl monomyristate) and two other derivatives (i.e., glucosyl and galactosyl monomyristates) were successfully synthesized from myristic acid and monosaccharides in two-step reactions. First, the myristic acid was converted to myristoyl chloride, and then the myristoyl chloride was reacted with fructose, glucose and galactose separately to produce the corresponding monosaccharide monomyristate derivatives. The structures of the synthesized products were confirmed by Fourier transform infrared (FTIR), proton and carbon nuclear magnetic resonance (^1^H- and ^13^C-NMR), and mass spectral (MS) data. The monomyristates esters were obtained in reaction yields of 45.80%–79.49%. The esters were then evaluated for their antimicrobial activity using the disc diffusion test. It was found that the esters exhibited a medium antibacterial activity against gram-positive bacteria; however, they showed a weak antibacterial activity against gram-negative bacteria. Amongst the esters, galactosyl myristate yielded the highest antibacterial activity against *Salmonella typhimurium*, *Staphylococcus aureus* and *Bacillus subtilis*, while glucosyl monomyristate exhibited the highest antibacterial activity only against *Escherichia coli*. Additionally, all products showed remarkable antifungal activity against *Candida albicans*. These findings demonstrate that monosaccharide monomyristate derivatives are promising for use as biocompatible antimicrobial agents in the future.

## 1. Introduction

Food contamination caused by microorganism activity has a serious effect on human health, particularly via digestive diseases [1,2]. The common harmful bacteria are *Staphylococcus aureus (S. aureus)*, *Escherichia coli (E. coli)*, *Bacillus subtilis (B. subtilis)* and *Salmonella typhimurium (S. typhimurium)*, while one example of common harmful fungi is *Candida albicans (C. albicans)* [3,4,5]. To prevent contamination, industries use antimicrobial agents to preserve their products by inhibiting microorganism activity. However, some antimicrobial agents are toxic and also non-biodegradable, thus raising other problems for human consumption [6].

Monosaccharide esters have attracted many researchers globally due to their biocompatibility [7,8]. They are specially developed in the biological and pharmaceutical fields for their antimicrobial, antioxidant, etc., properties [9,10,11,12,13,14,15]. Monosaccharide esters have usually been isolated from natural products or prepared through enzymatic reaction [16,17,18]. Li et al. successfully isolated sucrose fatty acid esters from *Astragalus membranaceus*, however, the isolation yield was less than 0.01% [16]. Enayati et al. reported that lactose fatty acid ester can be synthesized using immobilized *Candida antarctica* on Immobead 150, however, the purification of the product requires solvent extraction and high-performance liquid chromatography [17]. Both isolation and enzymatic process are time-consuming and expensive. Other reports have shown that carbohydrate esters have been successfully obtained in one-step synthesis between carbohydrate and acyl chloride using *N*,*N*-dimethylaminopyridine as the catalyst [18], although the yield was only 14%–58%, which is unfavorable for industrial applications.

Palm oil is one of the pivotal commodities because of its wide applicability in daily activities [19]. Palm oil contains both saturated and unsaturated fatty acids, such as palmitic, oleic, linoleic, stearic and myristic acids [20]. Myristic acid or tetradecanoic acid is a saturated fatty acid which has gained interest in organic synthesis, food industries and the pharmaceutical field as an antimicrobial, antioxidant and anticancer agent [21,22,23]. In our previous study, monomyristin derivatives of sugars show higher antibacterial activities against *S. aureus*, *E. coli*, *B. subtilis* and *Aggregatibacter actinomycetemcomitans* (*A. actinomycetemcomitans*). It was found that a 1-monomyristin derivative exhibits higher antifungal activity against *C. albicans* than a monopalmitin derivative [24]. Furthermore, the lauric esters of monosaccharide also exhibited medium antifungal and antibacterial activity against gram-positive bacteria [25]. Even though glucosyl and galactosyl monomyristates have been synthesized [26,27], their antimicrobial assay has yet to be evaluated.

Therefore, in the present work we synthesized the myristic acid ester derivatives of monosaccharides via the intermediate of myristoyl chloride. The synthesized esters included fructosyl, glucosyl and galactosyl monomyristate compounds. All the esters were evaluated for their antibacterial activity against gram-positive and gram-negative bacteria, as well as their antifungal activity.

## 2. Results and Discussions

### 2.1. Synthesis of Monosaccharide Monomyristate

**Figure 1:**
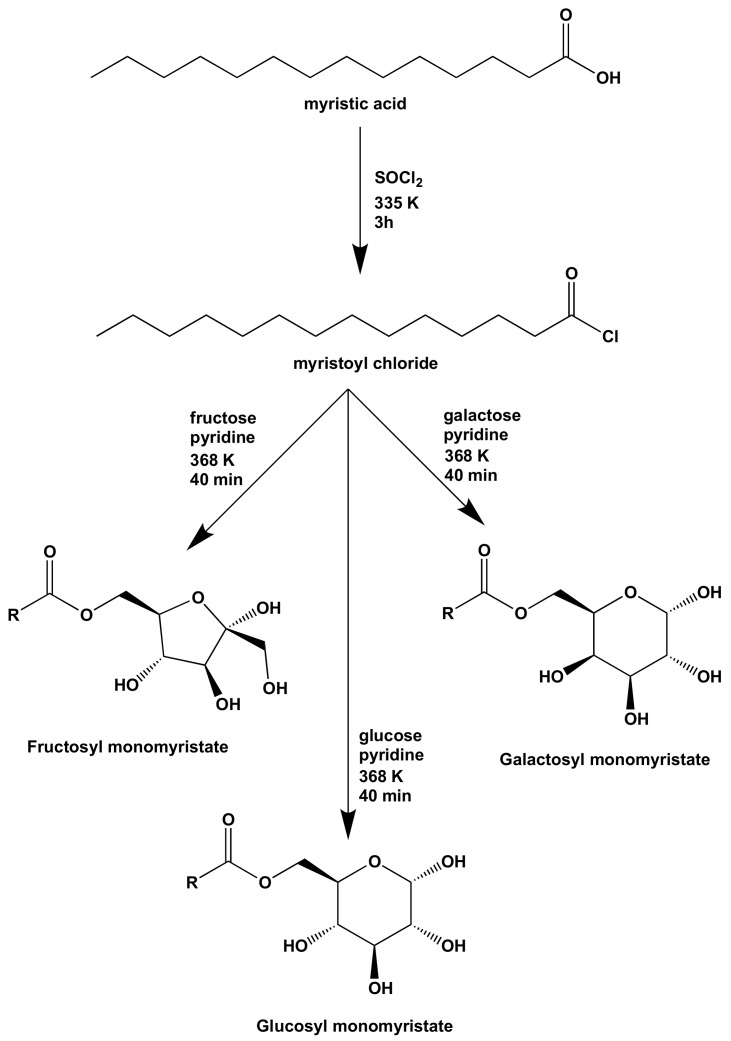
Reaction scheme of monosaccharide monomyristate derivatives from myristic acid (R = -C_13_H_27_) as the starting material.

The synthesis of monosaccharide myristic acid ester derivatives was carried out through myristoyl chloride as the intermediate as shown in Figure 1. The myristoyl chloride was prepared by reaction of myristic acid and thionyl chloride in a 1:2 molar ratio. The conversion of myristic acid to myristoyl chloride was employed to increase the reactivity for further esterification reaction. The Fourier transform infrared (FTIR) and mass spectra (MS) of the prepared myristoyl chloride were in agreement with those standard spectra of myristoyl chloride. The FTIR spectrum showed absorption peaks at 1797 and 570 cm^−1^ for C=O and C–Cl stretching, respectively. The gas chromatography (GC) chromatogram confirmed the purity of the product from the appearance of a single peak at retention time 31.5 min. Even though the MS spectrum did not show the molecular ion due to the high reactivity of the C–Cl bond, the fragment at *m*/*z* = 211 was observed due to the loss of the Cl radical from the molecular ion.

Myristoyl chloride was used as the reactant for esterification of the monosaccharide fructose, glucose and galactose to produce their myristoyl ester derivatives. The reaction was conducted in pyridine as the solvent, which facilitates the binding of the side product of hydrochloric acid. The fructosyl, glucosyl and galactosyl monomyristates were obtained in yields of 74.94%, 45.80% and 58.97%, respectively. The purity of the ester product was confirmed by the presence of a single peak in a GC chromatogram. The chemical structure of each ester was confirmed by spectroscopy analysis. The obtained FTIR, MS and nuclear magnetic resonance (NMR) spectra of glucosyl and galactosyl monomyristates in the present work were similar to the previous reports by Fregapane et al. and Baczko et al. [25,26], confirming that their synthesis was successfully carried out. Meanwhile, fructosyl monomyristate was found as the new compound in the present work. Alfindee et al. reported that the esterification of monosaccharide is a regioselective reaction due to the higher reactivity of the primary alcohol functional group [18]. Therefore, the FTIR and NMR spectra of the synthesized products are essentially similar to the spectra of the corresponding unreacted monosaccharide, except for the ester part of the myristoyl group. The FTIR spectra of the products showed the vibration of the C=O group of the ester moiety of the monosaccharide monomyristate derivatives at 1743–1751 cm^−1^. The ^1^H- and ^13^C-NMR spectra of fructosyl myristate, glucosyl myristate and galactosyl myristate are shown in Figure S1–S6. The ^1^H-NMR spectra of the products showed a pair of doublet of doublet signal at 4.33, 5.36 and 4.36 ppm for -COOCH_2_- protons of fructosyl-, glucosyl and galactosyl monomyristates, respectively. The resonance of the hydroxyl protons was not significantly different from that of the standard ^1^H-NMR spectra of the monosaccharide as the reactant, with -CH_2_-*O*- peaks shifted from 3.92 to 4.33–5.36 ppm, indicating that the esterification only occurred on the C-6 hydroxyl group. The carbonyl ester signals of these monomyristate esters were found at 174.1, 173.6 and 173.9 ppm, respectively, on their ^13^C-NMR spectra. These results confirm that the monosaccharide monomyristate products were successfully synthesized.

### 2.2. Antibacterial and Antifungal Activity Assays of Monosaccharide Monomyristate Derivatives 

The result of antibacterial and antifungal activity assays of the synthesized products are listed in Table 1. Compared to the positive control, all synthesized products exhibited medium antibacterial activity for the gram-positive bacteria, while the antibacterial activity for the gram-negative bacteria was insufficient. Galactosyl monomyristate yielded a 6.0-mm inhibition zone for *S. aureus*, while it did not show any antibacterial activity against *E. coli* and *S. typhimurium* at 0.50% concentration. This is probably due to the different cell wall composition between the gram-positive and gram-negative bacteria. The gram-positive bacteria have a simple cell wall structure, i.e., monolayer peptidoglycan, which contains 1%–4% lipid, while gram-negative bacteria have three-layer cell walls, i.e., lipoprotein, lipopolysaccharide and peptidoglycan, which contain 11%–12% lipid [28].

From Figure 1, it is reasonable that the synthesized products exhibit antibacterial activity to the gram-positive bacteria due to high polarity of the monosaccharide monomyristates. The presence of hydroxyl groups on the glycosyl part of the esters allows them to interact with the gram-positive bacterial cell wall. Once the compounds interact with the cell wall through intermolecular interaction, such as hydrogen bonding and van der Waals interaction, the cell will be destabilized, which can lead to bacteria lysis [28,29,30]. However, the prepared compounds also showed weak antibacterial activity to the gram-negative bacteria, perhaps due to the presence of the hydrophobic alkyl chain of the esters. From the results, it was found that galactosyl monomyristate exhibited the best antibacterial activity for *S. typhimurium* and the gram-positive bacteria, *S. aureus* and *B. subtilis* at 2.50%, 12.5% and 12.5% concentrations, respectively, while glucosyl monomyristate showed the highest antibacterial activity against *E. coli*.

In the antifungal assay, all synthesized compounds showed significant antifungal activity against *C. albicans* compared to the positive control. All the esters yielded the largest inhibition zones at 2.5% concentration. Similar to the predicted mechanism of action for antibacterial activity, the hydroxyl groups of the esters are also thought to interact with ergosterol on the fungi membrane cell, and thus can interfere with its function in maintaining membrane integrity [31]. The results of antibacterial assay showed that the pyranosyl derivatives (glucosyl and galactosyl monomyristates) revealed higher antibacterial activity than the furanosyl derivative (fructosyl monomyristate). In contrast, in the antifungal assay, the furanosyl derivative showed higher antifungal activity than the pyranosyl derivatives. This suggested that the stereochemistry of the glycosyl moieties can also influence the outcome of the antibacterial and antifungal activity. Table 1 clearly shows that the glucosyl ester exhibited higher antifungal activity than galactosyl ester at the same concentration.

A comparison of antibacterial and antifungal activities of the present work and previous reports is shown in Table 2. The ester fructose or glucose monocaprate showed higher antibacterial activity than the synthesized compounds, indicating that the alkyl chain length of the fatty acid could be critical. Compared to the monolaurate esters, the antimicrobial activity of the monomyristate esters against *S. aureus* is higher. A similar trend is also found for the antifungal activity, except for galactosyl monolaurate and galactosyl monomyristate. Glucosyl monomyristate shows higher antibacterial activity against *E. coli* than glucosyl monolaurate. However, monosaccharide monolaurates exhibit higher antibacterial activity against *B. subtilis*, except for glucosyl monolaurate. In general, it can be concluded that monosaccharide monocaprate showed higher antibacterial activity than monosaccharide monomyristate. Furthermore, the monosaccharide monomyristates have higher antibacterial and antifungal properties than the monosaccharide monolaurates. Even though the alkyl chain of caprate is lower than the laurate and much lower than the myristate, the order of their biological activity is not in a similar trend. Further experiment is required to know the effect of the alkyl chain length on the antimicrobial activity for the monosaccharide fatty acid derivatives.

Sucrose myristate and monomyristin yielded larger inhibition zones against *S. aureus* compared with the monosaccharide monomyristate, indicating that the number of hydroxyl groups is important for antibacterial activity against *S. aureus*. However, monosaccharide monomyristate showed higher antibacterial activity against *B. subtilis* compared with 2-monomyristin. Glucosyl monomyristate also exhibited higher antibacterial activity against *E. coli* compared with 1-monomyristin and sucrose myristate. Furthermore, all monosaccharide monomyristates exhibited significant antifungal activity compared with sucrose myristate and monomyristin compounds. These findings are valuable to develop potential antibacterial and antifungal agents from biodegradable and abundantly available raw materials.

## 3. Experimental

### 3.1. Materials

Myristic acid, D-(-)-fructose, D-(+)-glucose, D-(+)-galactose, anhydrous sodium sulfate, 4-isopropyl-3-methylphenol, pyridine, sabouraud 4% dextrose and brain heart broth were purchased from Merck (Darmstadt, Germany) in pro analytical grade, while 1 mol dm^−3^ thionyl chloride in dichloromethane was purchased from Tokyo Chemical Industry (Tokyo, Japan). Organic solvents such as dimethylsulfoxide (DMSO), methanol, chloroform and ethyl acetate were of analytical grade from Merck and used without any further purification.

### 3.2. Apparatus

The Fourier transform infrared (FTIR) spectra of the products were obtained from a FTIR spectrophotometer (Shimadzu Prestige 21, Tokyo, Japan). The purity and mass spectra of the synthesized products were characterized by gas-chromatography-mass spectrometer (GC-MS, Shimadzu QP 2010S). Meanwhile, the proton and carbon nuclear magnetic resonance (^1^H- and ^13^C-NMR) spectra of the products were recorded on a JEOL JNM-ECZ500R/S1 spectrometer (JEOL Ltd., Tokyo, Japan) employing tetramethylsilane as the internal standard in deuterated chloroform solvent.

### 3.3. Synthesis of Myristoyl Chloride

The synthesis of myristoyl chloride was started by melting myristic acid (0.7 g, 3.0 mmol) at 318 K and then thionyl chloride solution in dichloromethane (6.0 mL, 6.0 mmol, 2 equivalent) was added dropwise. The dichloromethane was removed by distillation and the temperature was maintained at around 335 K for 3 h. After the reaction completed, the excess of thionyl chloride was removed by distillation at 350 K. The chemical structure of the product was elucidated by FTIR and GC-MS spectrometry. The myristoyl chloride product was obtained as a clear light-yellow liquid in 93.24% yield. FTIR (KBr pellet method, cm^−1^): 2916 and 2854 (Csp^3^-H stretching), 1797 (C=O stretching), 1458 (-CH_2_- bending), 1373 (-CH_3_ bending), 570 (C-Cl stretching). GC: a single peak at 31.5 min retention time. MS (EI): m/z = 211 (M^+^-Cl, 10%), 166 (5%), 140 (7%), 126 (10%), 112 (24%), 98 (91%), 84 (73%), 57 (64%), 43 (base peak), 28 (40%).

### 3.4. Synthesis of Monosaccharide Monomyristate

Monosaccharide (0.2 g, 1.0 mmol) was dissolved in pyridine (1 mL, 12 mmol). Then myristoyl chloride (0.7 g, 3.0 mmol, 3 equivalent) was added dropwise and the mixture was heated at 368 K for 40 min. After the reaction completed, the mixture was neutralized with 10%* v*/*v* HCl. The desired product was extracted with chloroform and dried over anhydrous sodium sulfate. The organic solvent was evaporated, and the residue washed with methanol to obtain the desired product. The chemical structure of the product was elucidated by FTIR, GC-MS, ^1^H- and ^13^C-NMR spectrometry. Chemical structures of the monosaccharide monomyristate derivatives are shown in Figure 1. For the GC-MS analysis, the used column was Abdel 5MS (30 m long, 250 μm ID, and 0.25 μm film), while Helium gas was used as the mobile phase. The column oven temperature was 343 K and the injection temperature was 573 K. The total flow was 27.3 mL min^−1^ while the column flow was 0.49 mL min^−1^ with 25.5 cm s^−1^ linear velocity.

*Fructosyl monomyristate*. ((*2R,3S,4S,5S*)-3,4,5-trihydroxy-5-(hydroxymethyl)tetrahydrofuran-2-yl)methyl tetradecanoate. White solid in 79.49% yield. m.p: 43–44 °C. FTIR (KBr pellet method, cm^−1^): 3441 (OH stretching), 2916 and 2854 (Csp^3^-H stretching), 1743 (C=O stretching), 1465 (-CH_2_- bending), 1331 (CH_3_ bending), 1172 (C-O-C stretching). GC: a single peak at 38.1 min retention time. ^1^H-NMR (CDCl_3_, 500 MHz, ppm): 0.86 (t, 3H, CH_3_ myristate, *J* = 7.5 Hz), 1.23 (br s, 20H, -CH_2_- myristate), 1.60 (s, 2H, -CH_2_-CH_2_COO-), 2.32 (m, 2H, -CH_2_COO), 4.11-4.16 (m, 3H, CH-OH and CH_2_-OH), 4.33 (dod, 2H, COOCH_2_-, *J* = 7.0 Hz), 5.27 (t, 2H, -CH-OH, *J* = 6.0 Hz), 5.34 (t, 1H, CH_2_-CH-OH, *J* = 5.5 Hz). ^13^C-NMR (CDCl_3_, 125 MHz) δ (ppm): 14.3 (-CH_3_), 22.9 (-CH_2_-Me), 25.0+29.3+29.5+29.6+29.7+29.8+29.9+30.0+30.1+32.1+34.3 (11 -CH_2_-), 63.4 (-CH_2_-OH), 64.7 (-CH_2_-O-), 65.5+65.6+80.3 (3 -CH-OH), 80.4 (-CH-O-), 174.1 (C=O). MS (EI): m/z = 276 (M^+^-C_8_H_18_, 2%), 256 (M^+^-C_10_H_14_, 3%), 228 (25%), 211 (5%), 185 (38%), 171 (13%), 144 (28%), 129 (63%), 113 (18%), 97 (20%), 73 (C_3_H_5_O_2_^+^, 100%, base peak), 55 (75%), 43 (18%).

*Glucosyl monomyristate*. ((*2R,3S,4S,5R,6S*)-3,4,5,6-tetrahydroxytetrahydro-2H-pyran-2-yl)methyl tetradecanoate. Yellow-brown solid in 45.80% yield. m.p: 43-44 °C. FTIR (KBr pellet method, cm^−1^): 3448 (OH stretching), 2924 and 2854 (Csp^3^-H stretching), 1751 (C=O stretching), 1465 (-CH_2_- bending), 1373 (CH_3_ bending), 1165 (C-O-C stretching). GC: a single peak at 38.1 min retention time. ^1^H-NMR (CDCl_3_, 500 MHz, ppm): 0.86 (t, 3H, CH_3_ myristate, *J* = 7.5 Hz), 1.23-1.29 (m, 20H, -CH_2_- myristate), 1.57 (m, 2H, -CH_2_-CH_2_COO-), 2.28 (m, 2H, -CH_2_COO), 4.08-4.19 (m, 3H, CH-OH), 5.36 (dod, 2H, COOCH_2_-, *J* = 9.5 Hz), 5.13 (m, 1H, CH_2_-CH-OH), 6.32 (d, 1H, -O-CH-OH, *J* = 5.5 Hz). ^13^C-NMR (CDCl_3_, 125 MHz) δ (ppm): 14.5 (-CH_3_), 22.9 (-CH_2_-Me), 25.1+29.3+29.6+29.7+29.8+29.9+30.0+30.1+30.2+32.2+34.2 (11 -CH_2_-), 61.6 (-CH_2_-O-), 67.8+70.2+70.3 (3 -CH-OH), 73.1 (-CH-O-), 89.1 (O-CH-OH), 173.6 (C=O). MS (EI): m/z = 276 (M^+^-C_8_H_18_, 2%), 256 (M^+^-C_10_H_14_, 3%), 228 (23%), 200 (7%), 185 (37%), 171 (11%), 144 (13%), 129 (56%), 113 (9%), 97 (16%), 73 (C_3_H_5_O_2_^+^, 100%, base peak), 55 (67%), 43 (16%).

*Galactosyl monomyristate*. ((*2R,3R,4S,5R,6S*)-3,4,5,6-tetrahydroxytetrahydro-2H-pyran-2-yl)methyl tetradecanoate. White solid in 58.97% yield. m.p: 42–43 °C. FTIR (KBr pellet method, cm^−1^): 3464 (OH stretching), 2924 and 2854 (Csp^3^-H stretching), 1743 (C=O stretching), 1465 (-CH_2_- bending), 1373 (CH_3 _bending), 1172 (C-O-C stretching). GC: a single peak at 37.9 min retention time. ^1^H-NMR (CDCl_3_, 500 MHz, ppm): 0.88 (t, 3H, CH_3_ myristate, *J* = 7.0 Hz), 1.25-1.31 (m, 20H, -CH_2_- myristate), 1.62 (m, 2H, -CH_2_-CH_2_COO-), 2.37 (m, 2H, -CH_2_COO), 4.13-4.16 (m, 3H, CH-OH), 4.36 (dod, 2H, COOCH_2_-, *J* = 7.0 Hz), 5.29 (m, 1H, CH_2_-CH-OH), 5.36 (d, 1H, -O-CH-OH, *J* = 5.5 Hz). ^13^C-NMR (CDCl_3_, 125 MHz) δ (ppm): 14.1 (-CH_3_), 22.7 (-CH_2_-Me), 24.8+29.0+29.5+29.6+29.7+29.8+29.9+30.0+30.1+31.9+34.0 (11 -CH_2_-), 63.1 (-CH_2_-O-), 64.4+65.3+65.4 (3 -CH-OH), 79.2 (-CH-OH), 101.6 (O-CH-OH), 173.87 (C=O). MS (EI): m/z = 276 (M^+^-C_8_H_18_, 2%), 256 (M^+^-C_10_H_14_, 3%), 228 (20%), 199 (8%), 185 (38%), 157 (8%), 129 (58%), 113 (15%), 97 (18%), 73 (C_3_H_5_O_2_^+^, 100%, base peak), 55 (75%), 43 (16%).

### 3.5. Antibacterial and Antifungal Activity Assay

The antibacterial and antifungal activity assays were conducted to evaluate the biological activity of the synthesized product according to the method that has been previously described [24]. The bacteria used were *S. aureus* and *B. subtilis* for the gram-positive bacteria, and *S. typhimurium* and *E. coli* for the gram-negative bacteria, while *C. albicans* was selected for antifungal assay. Briefly, the antibacterial assay was carried out by dissolving brain heart broth (6.5 g) in distilled water (100 mL) as the medium of nutrient agar for bacteria. The medium was sterilized in an autoclave at 394 K under 15 psi pressure for 15 min. After the medium was ready to use, the bacteria were grown in the medium. Wells were made at 6.0 mm diameter and the desired concentration (in % wt/v) of the synthesized product in DMSO (50 μL) was introduced to the medium. The sample was incubated at 310 K for 24 h and the inhibition zone diameter was calculated from measuring the transparent area around the sample well. A quantity of 4-Isopropyl-3-methylphenol (1% in DMSO) and DMSO were used as the positive and negative controls, respectively. The antifungal activity assay was conducted in a similar manner as those for the antibacterial assay, except that the brain heart broth was replaced by a Sabouraud 4.00% of dextrose agar powder as the agar medium of nutrient agar for fungi.

## 4. Conclusions

Three monosaccharide fatty acid ester derivatives, i.e., fructosyl, glucosyl and galactosyl monomyristates were successfully synthesized from myristic acid and their corresponding monosaccharides in two-step reaction via myristoyl chloride as the intermediary. The chemical structure of the products was confirmed by spectroscopic data. The yields of esterification were 74.94%, 45.80% and 58.97%, respectively. The synthesized compounds exhibited medium antibacterial activity against the gram-positive bacteria but showed weak antibacterial activity against the gram-negative bacteria. The synthesized compounds revealed a significant antifungal activity against *C. albicans*. Furthermore, it was found that the pyranosyl monomyristate derivatives exhibited higher antibacterial activity than the furanosyl monomyristate derivative, while the opposite order was found for antifungal activity.

## Figures and Tables

**Table 1:** Antibacterial and antifungal activity of the monosaccharide monomyristate derivatives against gram-positive bacteria, gram-negative bacteria and fungi.

Compounds	Concentration (% *wt*/*v*)	Inhibition Zone (mm)				
		*Gram-Positive*			*Gram-Negative*	*Fungi*
		*S. aureus*	*B. subtilis*	*E. coli*	*S. typhimurium*	*C. albicans*
Fructosyl monomyristate	0.02	3.0	2.1	0.0	0.0	6.7
	0.10	3.2	3.0	0.0	1.1	7.4
	0.50	3.9	4.1	0.0	0.0	7.3
	2.50	4.9	4.1	0.0	1.1	8.8
	12.5	6.3	3.1	0.0	0.0	7.7
Glucosyl monomyristate	0.02	0.0	2.0	1.0	0.0	6.2
	0.10	0.0	2.1	1.3	0.0	7.4
	0.50	4.2	3.1	2.1	0.0	7.3
	2.50	5.2	4.0	2.4	1.1	8.7
	12.5	6.6	4.1	0.0	0.0	7.9
Galactosyl monomyristate	0.02	5.1	2.2	1.2	1.0	2.6
	0.10	6.0	3.1	0.0	0.0	4.0
	0.50	6.0	1.3	0.0	0.0	4.8
	2.50	7.3	3.2	1.6	1.3	5.7
	12.5	8.5	4.3	0.0	1.2	3.6
Positive control ^a^	1.00	11.6	8.8	10.2	5.3	24.2
Negative control ^b^	-	0.0	0.0	0.0	0.0	0.0

^a^ Positive control: 1.00% of 4-isopropyl-3-methylphenol in DMSO. ^b^ Negative control: DMSO.

**Table 2:** Comparison of antibacterial and antifungal activity of the monosaccharide monomyristate derivatives with previous reports.

Compounds in 0.5% *wt*/*v* Concentration	Inhibition Zone (mm)				
	*Gram-Positive*		*Gram-Negative*		*Fungi*
	*S. aureus*	*B. subtilis*		*E. coli*	*C. albicans*
Fructosyl monomyristate	3.9	4.1		-	7.3
Glucosyl monomyristate	4.2	3.1		2.1	7.3
Galactosyl monomyristate	6.0	1.3		-	4.8
Fructose monocaprate^a^ [26]	15.2	16.5		7.1	*
Glucose monocaprate^a^ [26]	12.9	16.2		6.6	*
Fructosyl monolaurate [25]	2.3	5.1		-	6.8
Glucosyl monolaurate [25]	-	1.1		-	4.8
Galactosyl monolaurate [25]	4.0	2.0		-	6.0
1-Myristoyl-sn-glycerol [24]	10.3	2.4		1.5	-
2-Myristoyl-sn-glycerol [24]	20.0	*		29.5	-
1-Palmitoyl-sn-glycerol [24]	-	*		-	-
Sucrose myristate [25]	10.0	*		-	-
Glucose monocaprate^a^ [26]	12.9	16.2		6.6	*

^a ^Concentration of the sample is 2.25 μmol/disc. *No data available.

## Supplementary Materials

The supplementary materials are available online.
