# Supplementary material for: Antibacterial and Antifungal Activity of Three Monosaccharide Monomyristate Derivatives
Source: Molecules. 2019 Oct 14;24(20):3692. doi: 10.3390/molecules24203692 (PMC6832165; doi:10.3390/molecules24203692)
Supplement: Supplementary file 1 [file molecules-24-03692-s001.pdf]

Supplementary Materials

Antibacterial and Antifungal Activity of Three Monosaccharide Monomyristate Derivatives

Jumina Jumina [1,*], Mutmainah Mutmainah[2], Bambang Purwono[3], Yehezkiel Steven Kurniawan[4,#] and Yana Maolana Syah[5]

[1] Department of Chemistry, Faculty of Mathematics and Natural Sciences, Universitas Gadjah Mada, Yogyakarta 55281, Indonesia. Email: jumina@ugm.ac.id
[2] Department of Chemistry, Faculty of Mathematics and Natural Sciences, Universitas Gadjah Mada, Yogyakarta 55281, Indonesia. Email: mutmainah@mail.ugm.ac.id
[3] Department of Chemistry, Faculty of Mathematics and Natural Sciences, Universitas Gadjah Mada, Yogyakarta 55281, Indonesia. Email: purwono.bambang@ugm.ac.id
[4] Department of Chemistry, Faculty of Mathematics and Natural Sciences, Universitas Gadjah Mada, Yogyakarta 55281, Indonesia. Email: yehezkiel.steven.k@mail.ugm.ac.id
[5] Faculty of Mathematics and Natural Sciences, Institut Teknologi Bandung, Bandung 40132, Indonesia. Email: yana@chem.itb.ac.id
# Present address: Ma Chung Research Center for Photosynthetic Pigments, Universitas Ma Chung, Malang 65151 and Indonesia. Email: yehezkiel.steven@machung.ac.id
* Correspondence: jumina@ugm.ac.id; Tel.: +62-2745-45188

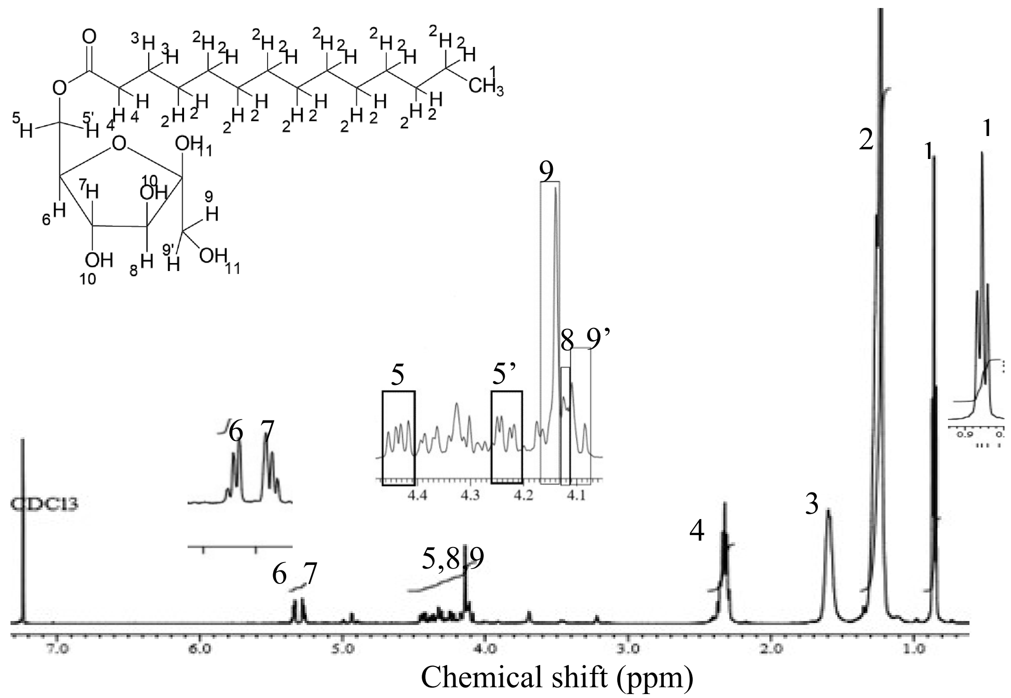

Figure S1. The ^{1}H-NMR spectrum of fructosyl myristate

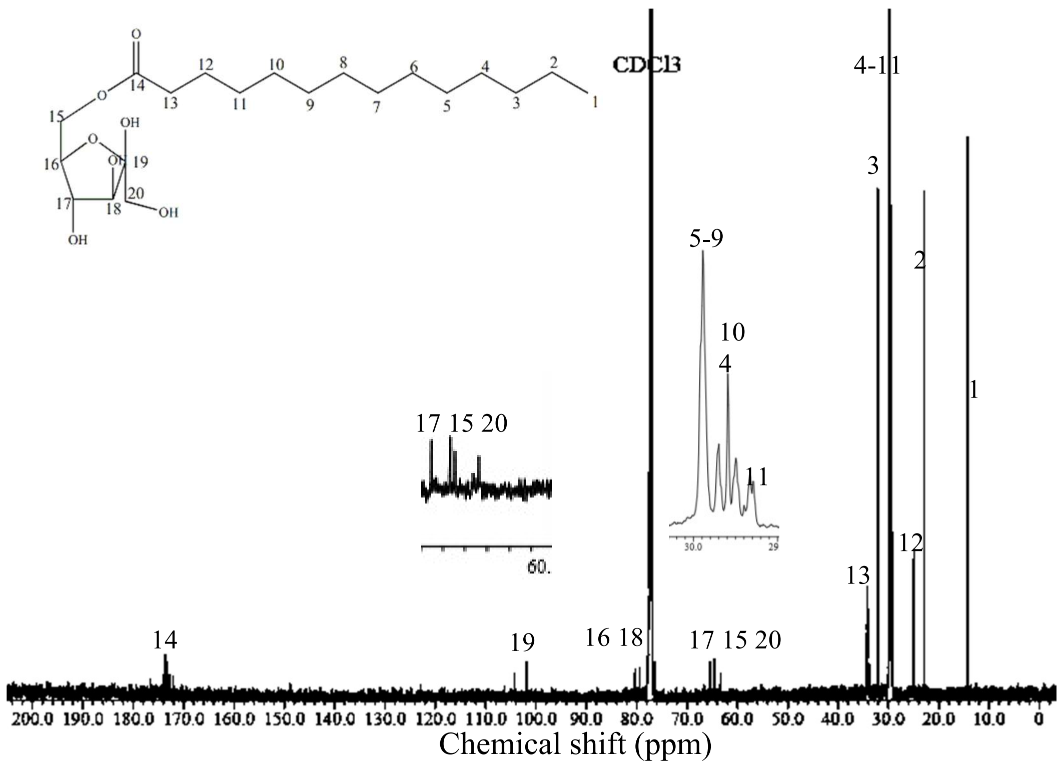

Figure S2. The ^{13}C-NMR spectrum of fructosyl myristate

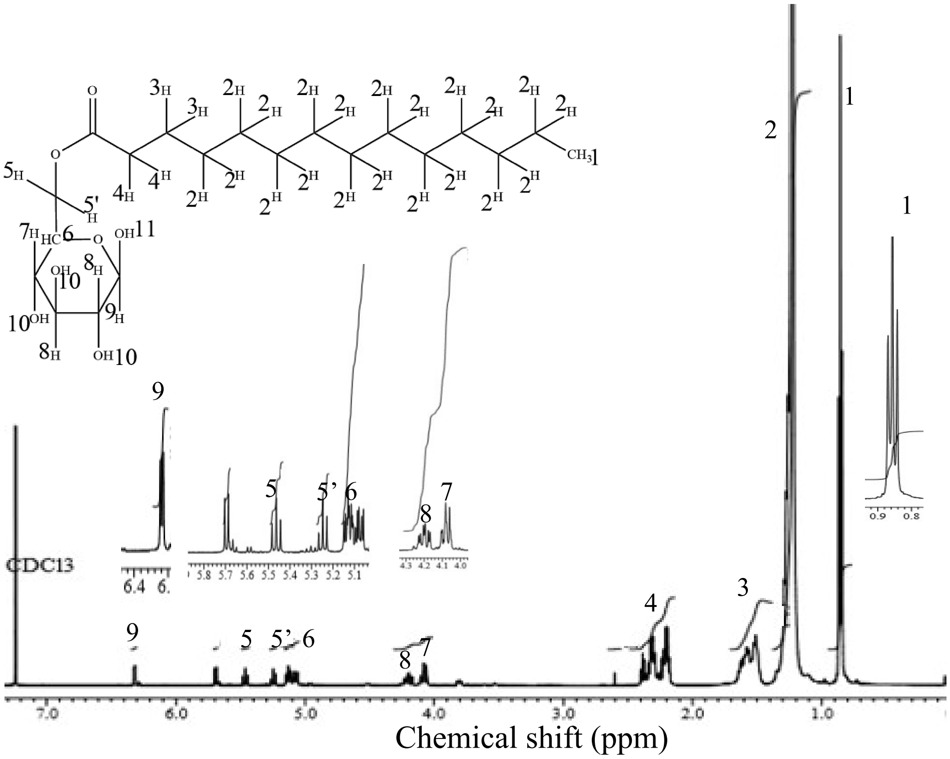

Figure S3. The ^{1}H-NMR spectrum of glucosyl myristate

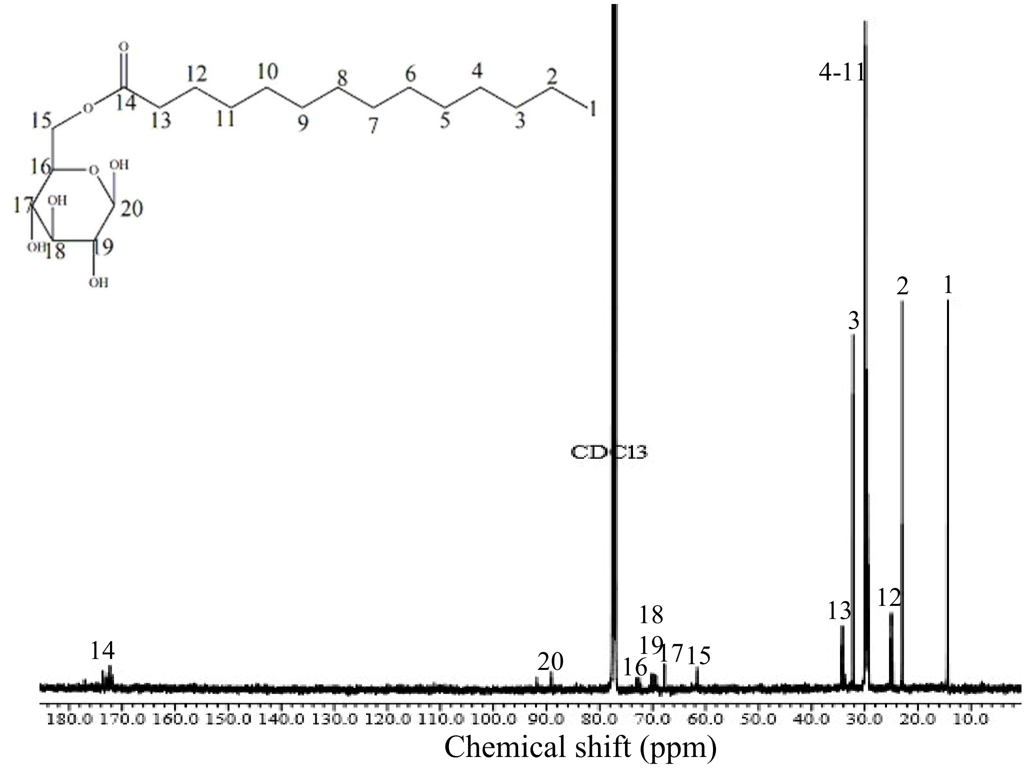

Figure S4. The ^{13}C-NMR spectrum of glucosyl myristate

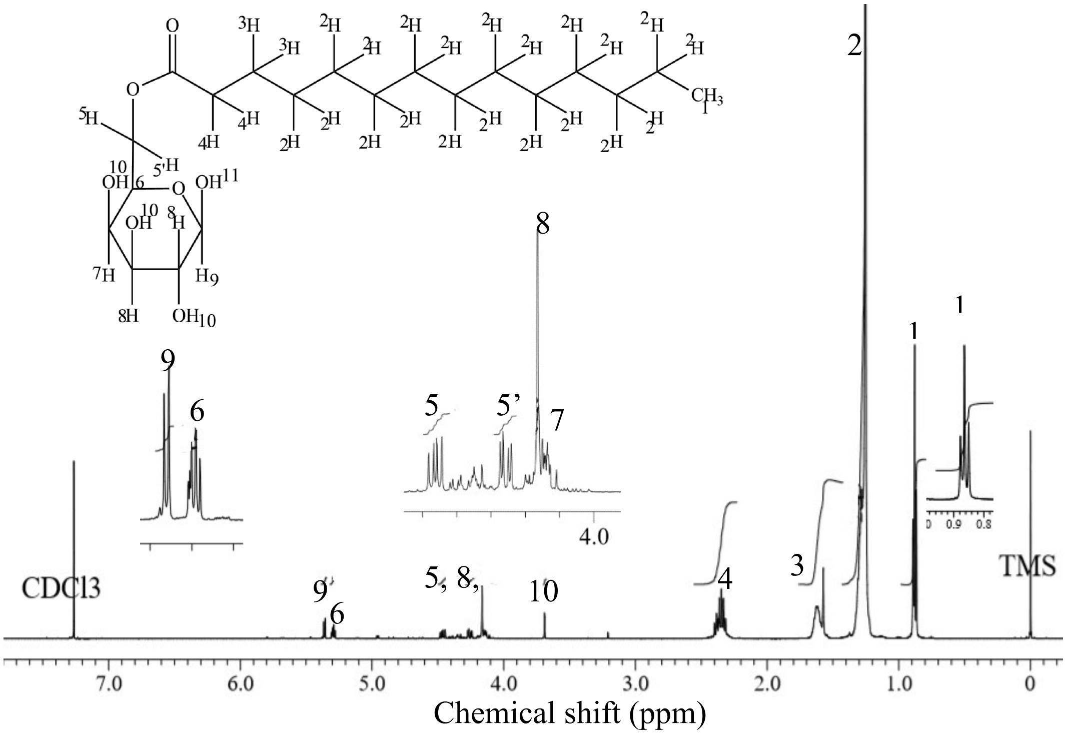

Figure S5. The ^{1}H-NMR spectrum of galactosyl myristate

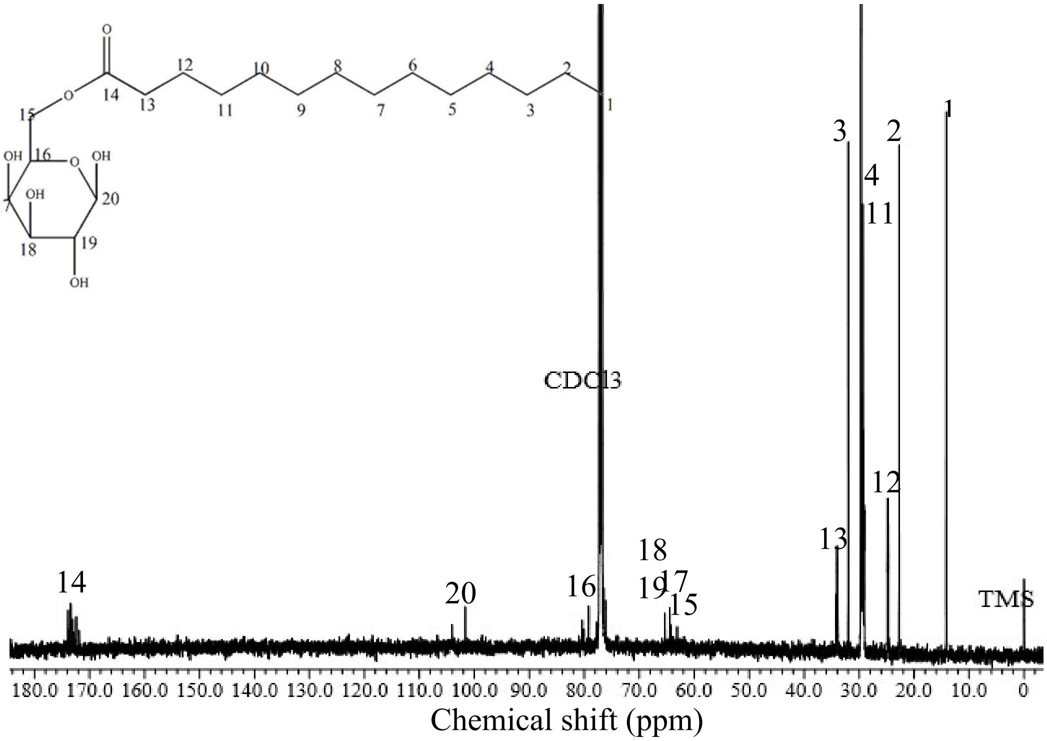

Figure S6. The ^{13}C-NMR spectrum of galactosyl myristate
